# Evaluation of Neonatologist-Provided Echocardiography in a Tertiary NICU in the United Arab Emirates

**DOI:** 10.7759/cureus.72551

**Published:** 2024-10-28

**Authors:** Syed Raza, Chokkiyil Ibrahim

**Affiliations:** 1 Department of Neonatology, Sheikh Shakhbout Medical City (SSMC), Abu Dhabi, ARE; 2 Department of Neonatology, Corniche Hospital, Abu Dhabi, ARE

**Keywords:** congenital heart disease, echocardiography in neonates, neonatal echocardiography, point-of-care ultrasound (pocus), tertiary nicu care

## Abstract

Aim: Our study aimed to review the safety profile of the echocardiography service provided by the neonatologist in our hospital. Until recently, echocardiography was the domain of paediatric cardiologists; today, neonatologists commonly use it to diagnose and manage newborns in many tertiary neonatal units. We achieved this by reviewing the indications and outcomes of neonatal echocardiography.

Method: We conducted a retrospective analysis of all neonatal echocardiograms performed between April 2013 and December 2016 at our institution. Data were collected on indications, echocardiographic diagnoses, and comparisons with findings from either a general paediatrician with cardiology expertise or a paediatric cardiologist from the regional referral hospital. Patients with minor diagnoses were followed locally, while those with critical congenital heart disease were referred to the regional centre for confirmation and further management. No follow-up was arranged for patients with normal cardiac anatomy, and their medical records were reviewed for any subsequent cardiac concerns post discharge.

Results: A total of 232 patients underwent 258 echocardiograms. Indications included asymptomatic heart murmur (35.7%), ruling out congenital heart disease and functional assessment (24%), follow-up/management of patent ductus arteriosus (14.7%), and presence of multiple congenital abnormalities (12.9%). Critical congenital heart disease was found in 12% of the cases, with 70% complete and 26% partial concordance with cardiologist reviews. Among non-critical cases (38.8%), 98% had findings concordant with follow-up echocardiograms. None of the patients with normal cardiac anatomy (42.7%) had post-discharge cardiac concerns within the first year of life.

Conclusion: Our results indicate that it is appropriate to provide cardiology services by a neonatologist, as findings were consistently compared and confirmed with those of trained paediatric cardiologists or specialists. It is also important to have regional referral unit support for advice and further management plans.

## Introduction

Echocardiography has become an established diagnostic tool in diagnosing and managing newborn infants in most tertiary neonatal units [1,2]. Traditionally, this domain belonged to paediatric cardiologists. Controversy still exists about whether a neonatologist should be performing echocardiography for structural echocardiography at the bedside without a certified qualification in echocardiography or whether they should restrict themselves to functional elements of the scan, taking into consideration only unique neonatal pathophysiology, which affects day to day cardiorespiratory management [3,4]. Due to resource constraints and working patterns, it is difficult to obtain round-the-clock on-site paediatric cardiology support for the vast majority of neonatal services across the globe. As a result, for the last two decades, neonatologists have increasingly taken on the role of performing bedside echocardiography, with several training and accreditation resources and standards for structural and functional imaging available to them [5,6].

Similar to the situation in many other parts of the world, most neonatal units in the United Arab Emirates do not have ready access to a paediatric cardiologist and have to rely on the skills of neonatologists to perform the initial echocardiographic assessment to guide immediate management. Cardiology support is usually provided with remote telemedicine tools, ad hoc visits, or transfer to a cardiology centre as required. In this paper, we describe the experience of providing a neonatologist-led echocardiography service at one of the tertiary neonatal centres in the city of Abu Dhabi, UAE.

## Materials and methods

We conducted a retrospective study in the neonatal unit at Mafraq Hospital (now renamed Sheikh Shakhbout Medical City), Abu Dhabi, UAE, covering the period from April 1, 2013, to December 31, 2016. Mafraq Hospital is a large tertiary hospital with a 26-bed neonatal intensive care unit (NICU) that admits approximately 2,200 patients per year and provides a full range of neonatal services, excluding neonatal cardiac surgery.

Bedside neonatal echocardiography in the NICU, and for babies referred from the postnatal ward, was performed by a single neonatologist trained and credentialed in the technique. During paediatric specialist training in the UK, this neonatologist completed six months in paediatric cardiology at Bristol Children's Hospital, followed by eight months at Freeman Hospital, Newcastle upon Tyne, as a paediatric cardiology specialist. After receiving Certificate of Completion of Training (CCT) training in paediatrics with a specialist interest in paediatric cardiology and neonatal medicine, the neonatologist has ensured ongoing continuous professional development (CPD) in neonatal echocardiography. This includes performing echocardiograms, planning management with paediatric cardiologists, and maintaining regular collaboration with the regional cardiology centre. Cardiology support was provided by the regional centre in the city, either through remote image review or by direct transfer to the centre if critical congenital heart disease was suspected based on the bedside echocardiography. For non-critical cardiac diagnoses, patients were either referred to the regional neonatal cardiology outpatient clinic or a local cardiology clinic run by a paediatrician with cardiology and echocardiography privileges.

Data were collected on all echocardiograms performed by the neonatologist from April 1, 2013, to December 31, 2016, with findings from either a general paediatrician with cardiology expertise or a paediatric cardiologist from the regional referral hospital. The collected data included basic demographic details such as birth weight, gestational age, and sex of the infants. Gestational ages were categorized as extreme preterm (<28 weeks), very preterm (28-31 + six weeks), moderate preterm (32-33 + six weeks), late preterm (34-36 + six weeks), and term (≥37 weeks).

For the study, the cardiac diagnoses were classified into four main groups as follows: group A - critical congenital heart disease, classified as cardiac lesions requiring intervention within the first year of life; group B - non-critical congenital heart disease, including atrial septal defect (ASD), ventricular septal defect (VSD), and patent ductus arteriosus (PDA); group C - functional cardiac problems, including poor function and pulmonary hypertension; group D - structurally normal, including small patent foramen ovale (PFO).

Comparisons of findings were made for infants transferred to the cardiac centre for immediate management or referred to the regional cardiac centre for follow-up care, to determine if management changed as a result of the cardiologists' imaging. Similar comparisons were also conducted for infants who were followed up locally by a paediatrician with cardiac imaging privileges without referral to the cardiology service.

No follow-up was arranged for patients with normal cardiac anatomy (group D), and their medical records were reviewed for any subsequent cardiac concerns post-discharge.

Data analysis was performed using Microsoft Excel (Microsoft Corporation, Redmond, WA) and results were displayed as summary statistics. Categorical data were presented as counts and percentages, while continuous data were summarized using medians and ranges due to variability in the data.

## Results

Over the study period, a total of 258 scans were performed on 232 infants. The infants spanned a wide gestational age and weight spectrum, half of them were preterm and the rest were term, as shown in Table 1.

**Table 1:** Demographic data (N = 232).

Category	Data
Weight (g), median (range)	2450 (460-4500)
Gestation (weeks), median (range)	38 (23-40)
Extreme preterm (<28 weeks), count (%)	42 (18%)
Very preterm (28-31 + 6 weeks), count (%)	26 (11%)
Moderate preterm (32-33 + 6 weeks), count (%)	21 (9%)
Late preterm (34-36 + 6 weeks), count (%)	26 (11%)
Term, count (%)	117 (50%)
Male-to-female ratio	124:108 (1.14:1)

The primary indications for performing echocardiograms during the study period are outlined in Table 2, which provides a detailed breakdown of the clinical scenarios that prompted the need for bedside imaging. These include the assessment of heart murmurs, critical cardiac screening failures, and the evaluation of neonates with congenital abnormalities or haemodynamic instability.

**Table 2:** Indications for neonatologist-performed echocardiogram.

Indication	Number of cases
Asymptomatic heart murmur	83
Failed critical congenital cardiac screen	2
Functional/structural assessment in a critically ill child	56
Follow-up/management of patent ductus arteriosus	34
Family history of congenital heart disease	4
To ascertain the central line position or presence of thrombus	3
Concerns on antenatal echo - structure/rhythm	20
To rule out congenital heart disease in the presence of other anomalies	30
Total	232

**Figure 1:**
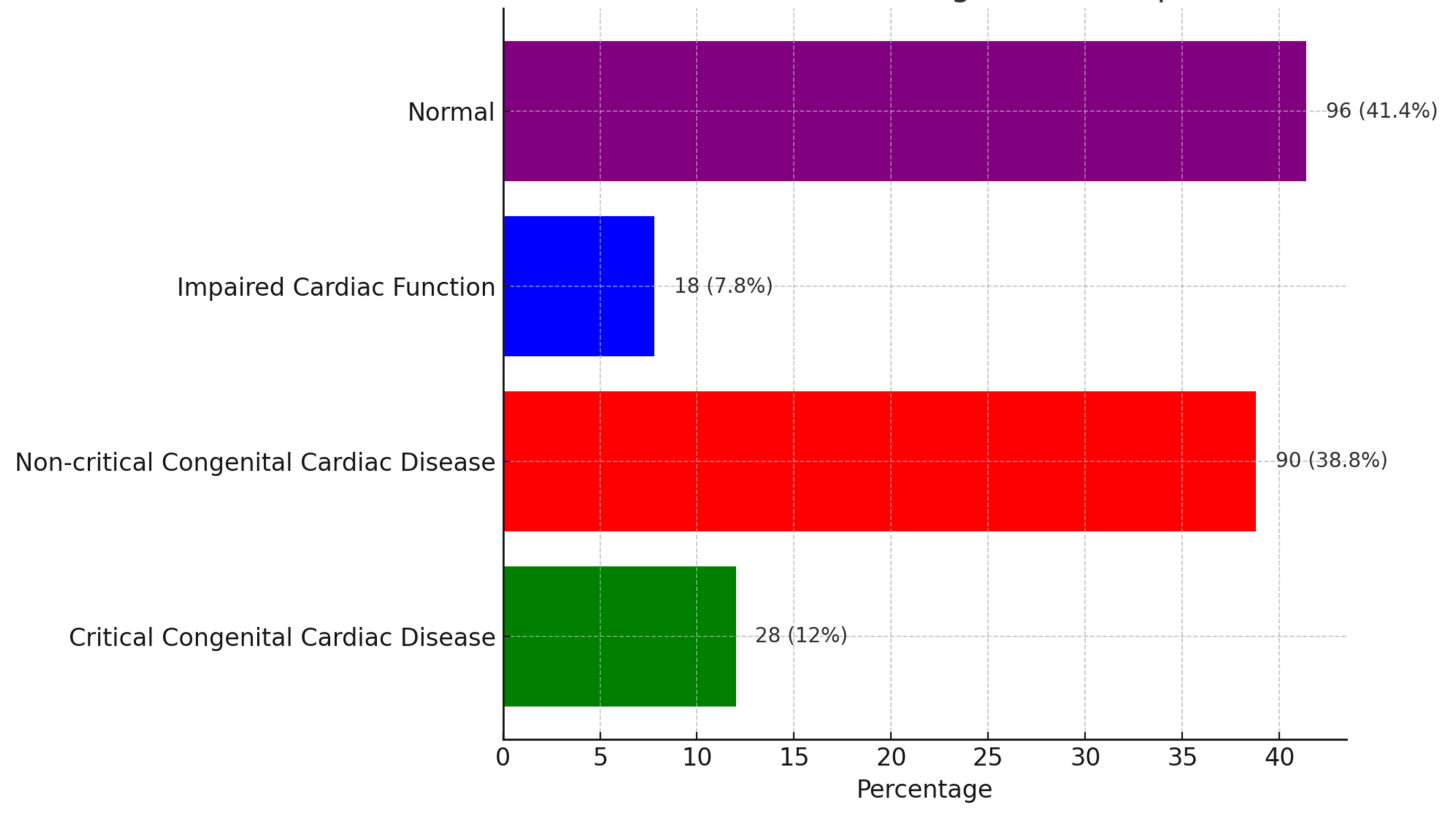
Distribution of patients according to diagnostic groups.

The distribution of diagnostic groups among the infants is summarized in Table 3, which categorizes the cases based on the severity and nature of cardiac conditions. Figure 1 illustrates the proportional distribution of these diagnostic groups, providing a clear visual representation of the data.

**Table 3:** Distribution of diagnostic groups.

Diagnostic group	Number of cases (%)
Group A: Critical congenital cardiac disease	28 (12%)
Group B: Non-critical congenital cardiac disease	90 (38.8%)
Group C: Impaired cardiac function	18 (7.8%)
Group D: Normal	96 (41.4%)

Group A: Critical congenital heart disease

**Figure 2:**
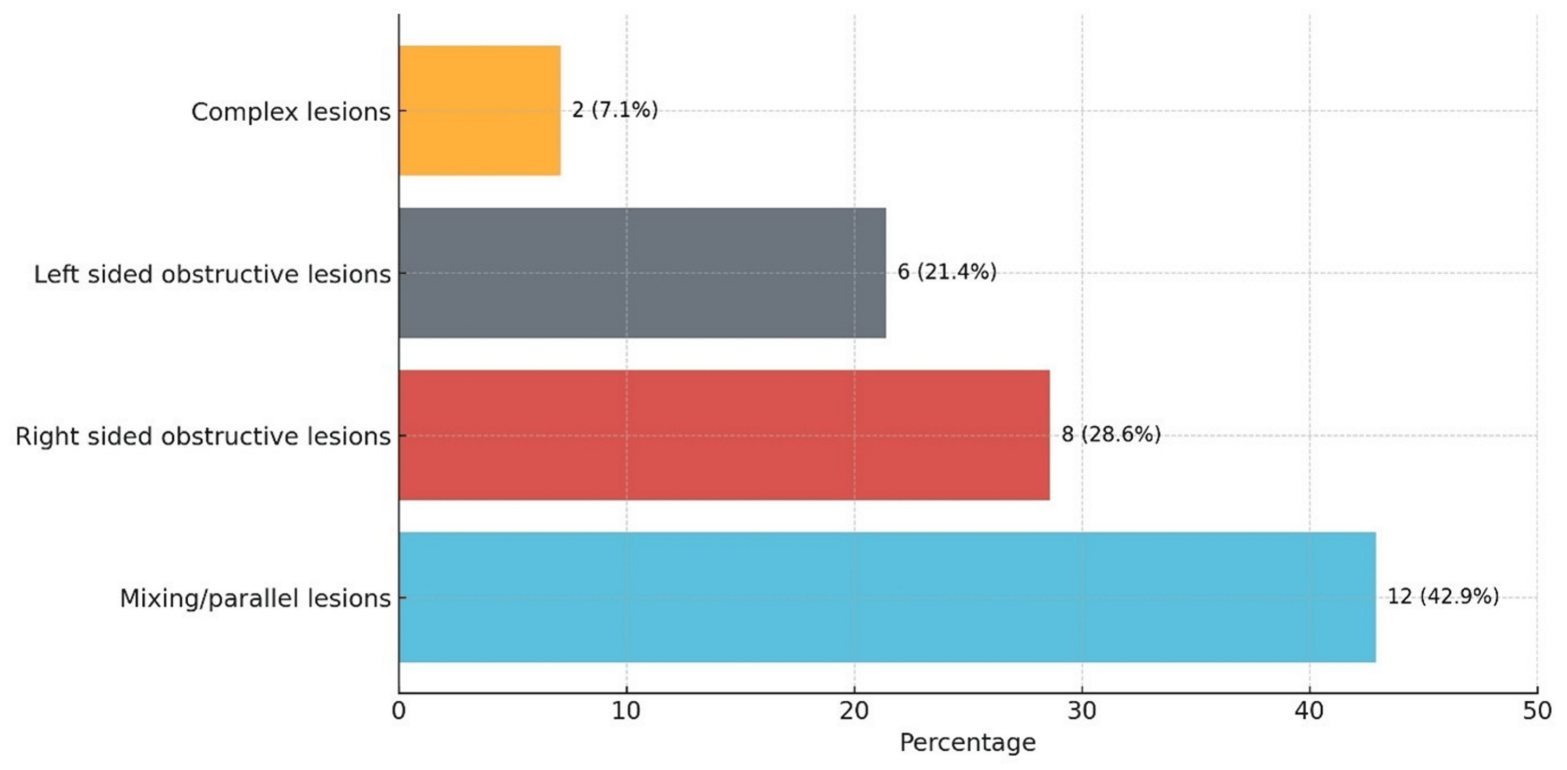
Distribution of lesion types in patients with critical congenital heart disease.

Critical congenital heart disease (CCHD) refers to severe heart defects present at birth that require intervention within the first year of life. The classification of the 28 infants with CCHD based on neonatologist-performed echo is represented in Figure 2 and details are in Table 4.

**Table 4:** Infants with suspected critical congenital heart disease - comparison between neonatology and cardiology scans. ASD: atrial septal defect; VSD: ventricular septal defect; CoA: coarctation of the aorta; PDA: patent ductus arteriosus; LV: left ventricle; HLHS: hypoplastic left heart syndrome; AS: aortic stenosis; PFO: patent foramen ovale; IAA: interrupted aortic arch; AVSD: atrioventricular septal defect; TA: tricuspid atresia; PS: pulmonic stenosis; TOF: tetralogy of Fallot; DORV: double outlet right ventricle; TAPVD: total anomalous pulmonary venous drainage; TGA: transposition of the great arteries; CHD: congenital heart disease; PMVSD: perimembranous ventricular septal defect; PA: pulmonary atresia.

Diagnosis on neonatologist-performed scan	Cardiologist confirmation	Concordance	Difference from neonatologist scan
Left-sided obstructed lesions			
ASD + VSD + CoA + PDA	Yes	Partial	Additional finding of a hypoplastic arch
Double inlet LV + PFO + PDA + hypoplastic arch/CoA	Yes	Complete	
LV rhabdomyoma	Yes	Complete	
HLHS + PFO + PDA	No - died at the base hospital	NA	
Peri-membranous VSD + critical AS + IAA	Yes	Partial	No aortic stenosis
VSD + CoA + HLHS + PDA	Yes	Complete	
Right-sided obstructive lesions			
AVSD + dextrocardia + TA	Yes	Partial	PA instead of TA
Ebstein's anomaly	Yes	Complete	
Moderate PS + AS	Yes	Complete	
Moderate pulmonary stenosis	Yes	Complete	
PS + ASD + PDA	Yes	Complete	
TOF with right-sided arch	Yes	Complete	
TOF with right-sided arch	Yes	Partial	Left-sided aortic arch instead of right-sided
TA + ASD + VSD	Yes	Complete	
Mixing lesions/parallel circulation			
AVSD	Yes	Complete	
ASD, PMVSD & PDA (AVSD)	Yes	Complete	
AVSD	Yes	Complete	
AVSD & dextrocardia	Yes	Complete	
AVSD + PDA	Yes	Complete	
DORV + peri-membranous VSD + ASD + PDA	Yes	Partial	Additional findings of CoA & hypoplastic arch
Pink TOF	Yes	Complete	
Single atrial cavity	Yes	Partial	Transitional type AVSD with single atrium and tiny primum VSD 1-2 mm
TAPVD	Yes	Complete	
TGA + peri-membranous VSD + PDA	Yes	Complete	
TGA + peri-membranous VSD + PDA	Yes	Complete	
TGA + ASD + peri-membranous VSD + PDA	Yes	Partial	No VSD
Complex lesions			
Complex CHD	Yes	Complete	
TA + IAA + VSD + PDA	Yes	Complete	

Infants with suspected CCHD were admitted to the NICU at Mafraq Hospital and commenced on appropriate cardiorespiratory support, including prostaglandin infusion when duct-dependent circulation was suspected. All but one child with suspected CCHD were transferred to the regional centre for a confirmatory scan and appropriate management. One child died prior to transfer and, therefore, could not be scanned by a cardiologist. Of the remaining 27 infants scanned, there was complete concordance between the findings of the neonatologist-performed and cardiologist-performed echocardiograms in 20 cases (74%), and partial concordance in seven cases (26%). However, the differences in findings did not affect the immediate management of any of the infants. All were either admitted to a cardiac intensive care unit for immediate management or discharged with follow-up and interventions later.

Group B: Non-critical congenital heart disease

Ninety (38.8%) of the 232 infants assessed had non-critical congenital heart disease, including ASD, VSD, PDA, and mild valvular stenosis. Initial management advice was based on the neonatologist-performed echocardiograms. The majority of these cases were in extreme preterm infants who were assessed for the diagnosis and management of PDAs. The rest involved well babies with a heart murmur or other congenital malformations, or critically ill infants who underwent echocardiography for haemodynamic assessment. If infants had a murmur or other concerns at the time of discharge, they were referred to the cardiology clinic for follow-up. Of the 90 infants, 55 (61%) had confirmatory or follow-up echocardiograms, either whilst still in the hospital or as outpatients, performed by either the paediatrician with cardiology privileges at Mafraq Hospital or the cardiology team at the regional referral centre. The summary of follow-up scans is presented in Table 5.

**Table 5:** Confirmatory follow up echocardiograms in infants with non-critical CHD (N = 55). CHD: congenital heart disease; VSD: ventricular septal defect; PS: pulmonic stenosis.

Follow-up scan done by	Number	Concordance with neonatologist scan	Details of discordant scans
Paediatrician with cardiology privileges	44	43 (98%)	Single atrium on neonatologist echo. Normal on follow-up
Paediatric cardiologist at the tertiary centre	4	4 (100%)	Nil
Both	7	6 (86%)	Perimembranous VSD on neonatologist echo. Additional finding of mild PS on follow-up

Only two of the 55 infants had different findings on follow-up echocardiograms. However, the discordance in findings did not affect the management or outcome of either infant.

Group C: Functional cardiac problems including poor function and pulmonary hypertension

Fifteen (6.5%) of the total infants were found to have persistent pulmonary hypertension or impaired cardiac function, despite having a normal structure on the neonatologist’s scan. The indication for the scan in these infants was haemodynamic and/or respiratory compromise, and all of them had persistent pulmonary hypertension of the newborn (PPHN). The majority (9/15) were term infants. The associated diagnoses are listed in Table 6. Management, including inotropes and pulmonary vasodilators, was initiated based on the echocardiography findings. Four of these infants had confirmatory scans by the paediatrician with cardiology privileges, with complete concordance of the findings.

**Table 6:** Associated diagnoses in infants with functional cardiac problems.

Associated diagnosis	Number (%)
Congenital diaphragmatic hernia	5 (33.3%)
Hypoxic ischaemic encephalopathy	2 (13.3%)
Meconium aspiration syndrome	1 (16.7%)
Prematurity	3 (20%)
Sepsis	3 (20%)
Suspected genetic abnormality	1 (16.7%)

Group D: Structurally normal heart

Ninety-nine (42.7%) of the infants were found to have a structurally normal heart with minor findings, including small PFOs, tiny closing PDAs in term infants within the first week, trivial atrioventricular valvular regurgitation, and functional peripheral pulmonic stenosis. The indications for the scans were either a heart murmur, investigation of unexplained respiratory symptoms, or screening for cardiac abnormalities in the presence of other extracardiac malformations. Infants from this group were referred for outpatient follow-up if a murmur was still present at discharge from the hospital. One-third of these infants (33/99) had follow-up appointments, four at the regional cardiology centre and the rest at the local paediatrician-run cardiology clinic. All of them had the same findings as the neonatologist’s scan or resolution of minor issues.

The linked electronic medical records of all the infants in groups B-D, who did not have a follow-up scan, were reviewed during the first year after discharge to see if any were admitted to the regional cardiac centre with a diagnosis of CCHD, which is the only centre that provides cardiac surgical and tertiary cardiac services. None of the patients in these groups had a delayed diagnosis of CCHD.

## Discussion

In this paper, we describe the experience of a single centre providing a neonatologist-delivered bedside echocardiography service in Abu Dhabi, UAE. It is now well-established that point-of-care (POC) echocardiography can be an important adjunct in the diagnosis and haemodynamic management of critically ill patients [3]. Echocardiography was traditionally the domain of cardiologists and echocardiography technicians. However, over the last three decades, neonatologists have increasingly taken on the responsibility of performing bedside echocardiography with cardiology support. Various models of this service have evolved worldwide, depending on local needs and the availability of resources [7-10]. There have been debates about whether a neonatologist should perform a structural assessment of the heart or focus purely on haemodynamic assessment to guide clinical management.

Most neonatal units worldwide do not have access to an onsite paediatric cardiologist around the clock [11]. As a result, neonatologists have increasingly taken on the role of performing the initial echocardiogram, even when a structural heart lesion is suspected, with support from cardiologists through telemedicine tools and ad hoc or scheduled visits [2]. In the past, neonatologists' training in this technique was largely informal, consisting of on-the-job training during their attachments in paediatric cardiology or under the guidance of other neonatologists skilled in neonatal echocardiography [12,13]. However, there has been a growing movement towards formalizing neonatologists' training in echocardiography to ensure patient safety and quality assurance [3-6]. Australian guidelines on POC neonatal echocardiography focus more on functional assessment, with less rigorous training required for structural assessment [14]. This approach diverges from the more recent European and North American guidelines, which emphasize the importance of ruling out any structural heart lesions as a prerequisite to assessing function [5,6].

Several institutions worldwide have published their experiences and current practices regarding neonatologist-performed echocardiography (NPE) in their respective geographical regions in recent years [7-10]. Most of the published literature on NPE focuses on the functional assessment of cardiovascular status in critically ill newborns and the assessment of PDA in preterm infants while emphasizing that structural scans remain the domain of paediatric cardiologists [8,9]. Researchers have examined the anatomic concordance between scans performed by trained neonatologists and cardiologists [2,15]. In a North American study by Bischoff et al., a 97.9% concordance rate was found between the anatomical findings of scans performed by neonatologists and those subsequently done by cardiologists [15]. Similar findings were reported in a study from the UK by Moss et al. [2].

In this study, we aimed to assess the safety of an NPE service in a region where healthcare infrastructure is rapidly evolving. As in many parts of the world, the majority of neonatal departments, including ours, do not have onsite paediatric cardiology services. Therefore, the immediate diagnosis of congenital cardiac disease, especially in critically ill newborn infants, depends on the availability of echocardiography skills among neonatologists. At the time of data collection for this study, there was only one neonatologist with the necessary skills in our unit. Of the 232 infants included in this study, 115 (49%) had confirmatory echocardiograms performed by either a paediatric cardiologist or a paediatrician with cardiology privileges. There was a high rate of anatomical concordance between the echocardiograms performed by the neonatologist and those performed by the cardiologist or paediatrician. Most importantly, 26 of 27 scans conducted by the neonatologist on infants with suspected CCHD showed complete or partial concordance with the cardiologist’s findings. No CCHD was missed in any of the neonatologist-performed scans, based on the review of unified electronic medical records for all infants. Thus, it can be assumed that the echocardiography service provided by neonatologists at our centre is safe for immediate management purposes.

Our study does have limitations. There are several organizations, including private institutions, within the state that provide paediatric services. Their medical records are not unified with the systems in the government sector, like ours. Therefore, if an infant with CCHD was missed at our centre and later admitted to one of these hospitals after discharge, we may not have captured them. However, only one centre in the city provides tertiary cardiac and cardiothoracic surgery services for children, and its medical records are unified with ours. Thus, we assume that any infants admitted to this centre would have been captured.

In summary, we have demonstrated the safety of a neonatologist-delivered echocardiography service in a tertiary neonatal unit, consistent with previously published literature. Given the limited availability of tertiary paediatric cardiac services in most healthcare systems, this is a safe model for practice. However, it must be implemented within a robust framework of training and quality assurance, as outlined in consensus statements from relevant professional bodies.

## Conclusions

Our findings demonstrate that a neonatologist-led echocardiography service within a tertiary neonatal unit is a safe and effective model for the diagnosis and management of both critical and non-critical cardiac conditions in neonates. The high concordance rate between the neonatologist and the cardiologist supports further dependability of the approach. This may fill important neonatal care gaps with appropriate training and support from regional cardiology centres, in particular, areas where the on-site paediatric cardiology expertise is small. This model has the potential to improve neonatal cardiac care worldwide while maintaining safety and quality.
